# ^18^F-Florbetaben PET beta-amyloid binding expressed in Centiloids

**DOI:** 10.1007/s00259-017-3749-6

**Published:** 2017-06-22

**Authors:** Christopher C. Rowe, Vincent Doré, Gareth Jones, David Baxendale, Rachel S. Mulligan, Santiago Bullich, Andrew W. Stephens, Susan De Santi, Colin L. Masters, Ludger Dinkelborg, Victor L. Villemagne

**Affiliations:** 1 Department of Molecular Imaging & Therapy, Centre for PET, Austin Health, Studley Road Heidelberg, Melbourne, VIC 3084 Australia; 2 Department of Medicine, University of Melbourne, Parkville, Melbourne, 3010 Australia; 3 eHealth, CSIRO Health and Biosecurity, Brisbane, QLD Australia; 4 Piramal Imaging GmbH, 13353 Berlin, Germany; 5 Piramal Pharma, Inc, Boston, MA 02108 USA; 6 The Florey Institute of Neuroscience and Mental Health, Melbourne, VIC 3052 Australia

**Keywords:** Florbetaben, Centiloid, Amyloid imaging, Standardization, Alzheimer’s disease

## Abstract

**Purpose:**

The Centiloid (CL) method enables quantitative values from Aβ-amyloid (Aβ) imaging to be expressed in a universal unit providing pathological, diagnostic and prognostic thresholds in clinical practice and research and allowing integration of multiple tracers and methods. The method was developed for ^11^C-PiB scans with zero CL set as the average in young normal subjects and 100 CL the average in subjects with mild Alzheimer’s disease (AD). The method allows derivation of equations to convert the uptake value of any tracer into the same standard CL units but first requires head-to-head comparison with ^11^C-PiB results. We derived the equation to express ^18^F-florbetaben (FBB) binding in CL units.

**Methods:**

Paired PiB and FBB PET scans were obtained in 35 subjects. including ten young normal subjects aged under 45 years (33 ± 8 years). FBB images were acquired from 90 to 110 min after injection. Spatially normalized images were analysed using the standard CL method (SPM8 coregistration of PET data to MRI data and the MNI-152 atlas) and standard CL regions (cortex and whole cerebellum downloaded from http://www.gaain.org).

**Results:**

FBB binding was strongly correlated with PiB binding (*R* ^2^ = 0.96, SUVR_FBB_ = 0.61 × SUVR_PiB_ + 0.39). The equation to derive CL values from FBB SUVR was CL units = 153.4 × SUVR_FBB_ − 154.9. The CL value in the young normal subjects was −1.08 ± 6.81 for FBB scans compared to −0.32 ± 3.48 for PiB scans, giving a variance ratio of 1.96 (SD_FBB CL_/SD_PiB CL_).

**Conclusions:**

^18^F-FBB binding is strongly correlated with PiB binding and FBB results can now be expressed in CL units.

## Introduction

There is an urgent need to standardize the results of quantitative measurements of Aβ-amyloid (Aβ) tracer binding measured with positron emission tomography (PET) in a way that permits integration of results from different tracers and different analysis methods and in a way that is readily accessible to scanning sites worldwide. Currently, there is wide variability in the numbers and methods used to report quantitative measures from Aβ scans [1] and results vary for each of the available tracers due to differences in both their specific and nonspecific binding properties and recommended reference regions [2–5]. Results are also influenced by the timing of scan acquisition after administration of the Aβ tracer, duration of the acquisition, image reconstruction algorithms, partial volume correction, choice and extent of cortical regions, and the quantitative analysis method used [6–16]. Consequently imaging laboratories must derive a normal range for their method and each Aβ radiopharmaceutical or rely on subjective visual reading.

This lack of consistency in image analysis methods and highly variable expression of the results impedes the pooling of data across sites and comparison of studies [17]. Lack of standardization prevents the application of universal cut-off values for diagnostic and prognostic purposes [18]. It also limits comparison of the relative effectiveness of therapies that claim to reduce Aβ burden [17], and limits comparison of the relative merits of Aβ tracers including in-vivo affinity for human Aβ and measurement variance. These limitations are important when considering the need for earlier detection of plaque and the measurement of changes over time and the effects of treatment.

An international working party of Aβ imaging researchers has developed a method to standardize quantitative Aβ imaging measures by scaling the outcome to the Centiloid (CL) scale [18]. This scale has a zero CL point that corresponds to the mean result obtained from scans in young adults who, based on age, are reasonably assumed to be free of Aβ plaques. The 100 CL point corresponds to the mean result of scans performed in a group of patients with typical Alzheimer’s disease (AD) of mild severity, the time when Aβ burden peaks in the course of AD [19, 20]. Consequently, the measurement units for this scale have been named to reflect the 100-point scale and the application to amyloid; hence the term “Centi-loid”. The CL method also allows comparison of tracer characteristics relative to PiB under strictly controlled head-to-head conditions.

**Fig. 1:**
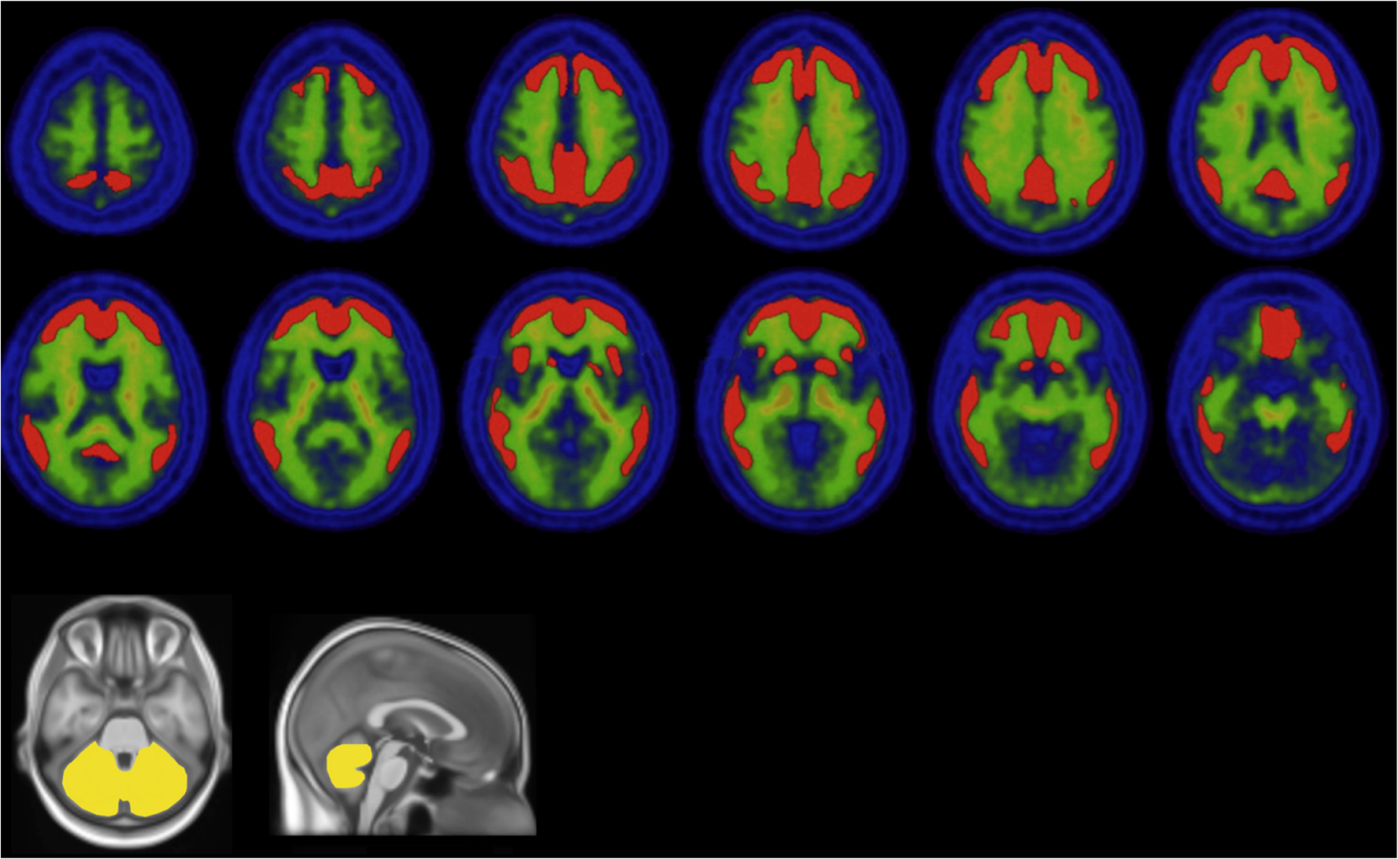
The standard Centiloid method cortical volume of interest (*red*) and the whole cerebellum reference region (*yellow*) normalized to MNI-152 space (adapted from Klunk et al. [18])

The data required to convert PiB PET standardized uptake value ratios (SUVR) to CL units is available from the Global Alzheimer’s Association Interactive Network (GAAIN) website (http://www.gaain.org). The website provides free access to a standard cortical volume of interest (VOI) that covers the areas of significant PiB Aβ tracer binding in AD and a whole cerebellum VOI to use as the reference region (Fig. 1). The linear equation required to convert the PiB SUVRs obtained by the standard CL method on any PiB scan acquired from 50 to 70 min after injection is supplied. A validation set of PiB and MRI scans is also supplied so that the user can confirm correct application of the method locally. It is then possible to derive an additional linear equation to convert the results obtained from any preferred in-house analysis method to CL units by analysis of the same PiB images that have been analysed by the standard CL method [18].

To perform the conversion from SUVR to CL for tracers other than PiB such as ^18^F-florbetaben (FBB), one site must first obtain matching PiB and FBB scans from the same individuals according to the standard method described by Klunk and colleagues [18]. By applying the standard CL method to these scans, a linear conversion equation (scaling factor) is derived that can then be used to express FBB in CL units. This data is then made available through GAAIN so that other sites can validate their application of the CL method to FBB images and then convert their own FBB scans to CL units, without the need to perform matching PiB scans, by applying the scaling factor described in this manuscript. Furthermore, the site can then apply their local analysis method and derive a second scaling factor that permits their usual in-house analysis method to provide results in CL units [18]. Provided this shows high linear correlation with the standard CL method, there is then no need to further use the standard method to provide results in CL.

**Fig. 2:**
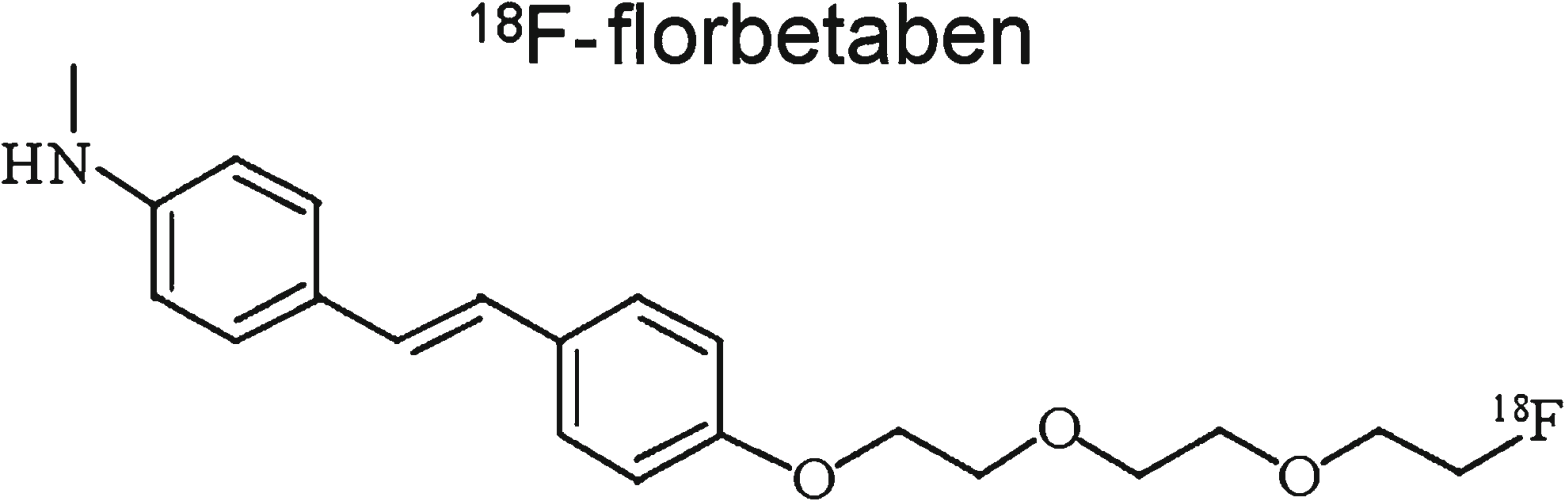
Chemical structure of ^18^F-florbetaben

FBB (*trans*-4-(*N*-methyl-amino)-4″-{2-[2-(2-[^18^F]fluoro-ethoxy)ethoxy]-ethoxy} stilbene; [^18^F]AV-1, [^18^F]BAY-94-9172; Neuraceq®) Fig. 2) was synthesized by Kung and colleagues and developed by Bayer Healthcare and Piramal Imaging. FBB has high affinity and specificity for Aβ [21], lack of binding to Lewy bodies or neurofibrillary tangles in post-mortem tissue at low nanomolar concentrations, and excellent correlation with global PiB retention [22]. FBB was the first reported ^18^F-labelled tracer to show a robust capacity to distinguish subjects with AD from those with other dementias and healthy elderly individuals [3, 23, 24]. FBB was also able to detect the presence or absence of AD pathology in a mixed population of cognitively impaired subjects [25, 26]. In patients with mild cognitive impairment (MCI), FBB binding was correlated with episodic memory and showed high accuracy for predicting conversion to AD during a follow-up of 4 years [25, 26]. A multicentre phase 3 trial confirmed that FBB is able to detect cortical fibrillar Aβ plaques, as assessed by visual reading, with 100% sensitivity and 92% specificity in relation to post-mortem silver staining [27]. FBB received European Medicines Agency (EMA) and Food and Drug Administration (FDA) approval for clinical use in February and March 2014, respectively.

In this report we describe the acquisition of the data and the derived linear equation required to convert FBB SUVRs to CL units. We also provide data on the relative performance of FBB and PiB in the same individuals using the standard CL methods.

## Materials and methods

The study was approved by the Austin Health Human Research Ethics Committee, and all subjects gave written informed consent.

### Subjects

Paired PiB and FBB PET scans were obtained in 35 subjects. The cohort comprised 10 healthy young controls aged under 45 years (33 ± 8 years) recruited specifically for this study, 6 subjects from a previously published cohort, and 19 subjects recruited specifically for this study who included 6 healthy elderly controls (71.3 ± 8 years, Mini-Mental State Examination, MMSE, score 29 ± 1), 9 patients with MCI ( 72 ± 5 years, MMSE score 28 ± 2), 8 patients with mild AD (69 ± 6 years, MMSE score 23 ± 3) and 2 patients with frontotemporal dementia (74 ± 8, MMSE score 23 ± 1). The demographics of the cohort are shown in Table 1. All subjects who had PiB and FBB PET studies within 3 months and on the same PET camera at the study site were included in this study.

**Table 1:** Demographics of all 35 subjects included in the study

Group	Number of subjects	Age (years)	MMSE score
Young healthy controls	10	33 ± 8	>28
Elderly healthy controls	6	71.3 ± 8	29 ± 1
Mild cognitive impairment	9	72 ± 5	28 ± 2
Alzheimer’s disease	8	69 ± 6	23 ± 3
Frontotemporal dementia	2	74 ± 8	23 ± 1

### Scanning

The paired PiB and FBB PET scans for each individual were obtained within 3 months of each other and with a minimum of 2 h between scans if PiB PET was done first or 24 h if FBB PET was done first. The scans obtained from the six previously reported subjects were acquired on a Philips Allegro PET camera in 3D mode and processed with rotating Cs-137 point source attenuation correction. The scans obtained in the other 29 subjects specifically for this study were all acquired on a Philips TF64 PET/CT scanner with CT attenuation correction. Images were reconstructed using a 3D row-action maximum likelihood algorithm (RAMLA) for the Allegro images and a line of response RAMLA for the TF64 images. Time of flight and resolution recovery reconstruction options were not used.

**Fig. 3:**
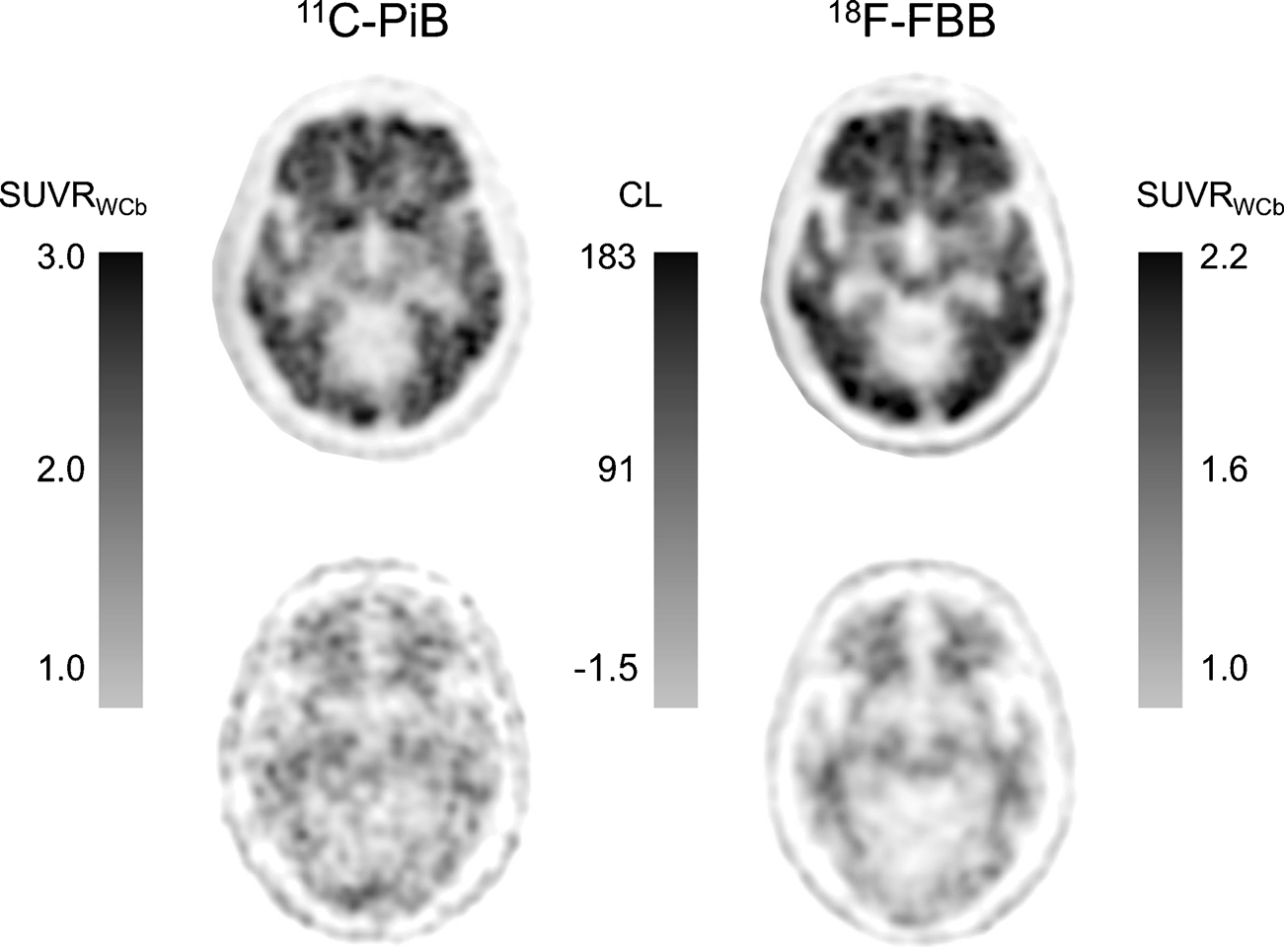
^11^C-PiB and ^18^F-FBB images in the same patient with mild AD (*top*) and the same healthy young control subject (*bottom*). The scales are the SUVR in relation to the whole cerebellum as reference region (SUVR_WCb_) and Centiloid (CL) units

Subjects were injected with 555 MBq (±10%) of ^11^C-PiB and 300 MBq (±10%) of ^18^F-FBB. In accordance with the standard CL protocol, the PiB acquisition was from 50 to 70 min after injection. FBB images were acquired from 90 to 110 min after injection in accordance with the manufacturer’s recommendation. Examples of matched images with the two tracers in a patient with mild AD and a young healthy subject are shown in Fig. 3 together with both SUVR and CL units.

MRI was performed in all subjects on a Siemens 3-T Trio camera. The T1 MP-RAGE sequence with 1 × 1 × 1.2 mm voxels was used for image registration. Partial volume correction was not performed.

### Image analysis

Each subject’s MRI image was coregistered to the MNI-152 template with SPM8 and then each subject’s PET image was coregistered via the derived MRI transformation parameters using the SPM8 unified segmentation method, as described in detail in the CL methodology paper [18]. The standard CL cortical and whole cerebellum reference VOIs were downloaded from the GAAIN website (Fig. 1) and applied to each scan after registration to the MNI-152 template. The local processing pipeline was first validated on the standard PiB image set from the GAAIN website. Then the paired PiB and FBB images were analysed with the standard method and CL templates from GAAIN to derive SUVRs that were plotted against each other. This produced the linear equation required to convert the standard method FBB SUVR to the equivalent or “calculated” PiB SUVR. (^PiB-Calc^SUVR): FBB SUVR = *m* × (^PiB^SUVR) + *b*; or ^PiB-Calc^SUVR = (FBB SUVR − *b*)/*m*. The equation to directly convert FBB SUVRs to CL units was derived by plotting FBB SUVRs against the CL units derived via conversion to ^PiB-Calc^SUVR. The mean and variance of PiB and FBB CL units were compared in the young normal adults and the variance ratio was obtained by dividing the standard deviation (SD) of the FBB CL value by the SD of PiB CL value.

## Results

**Fig. 4:**
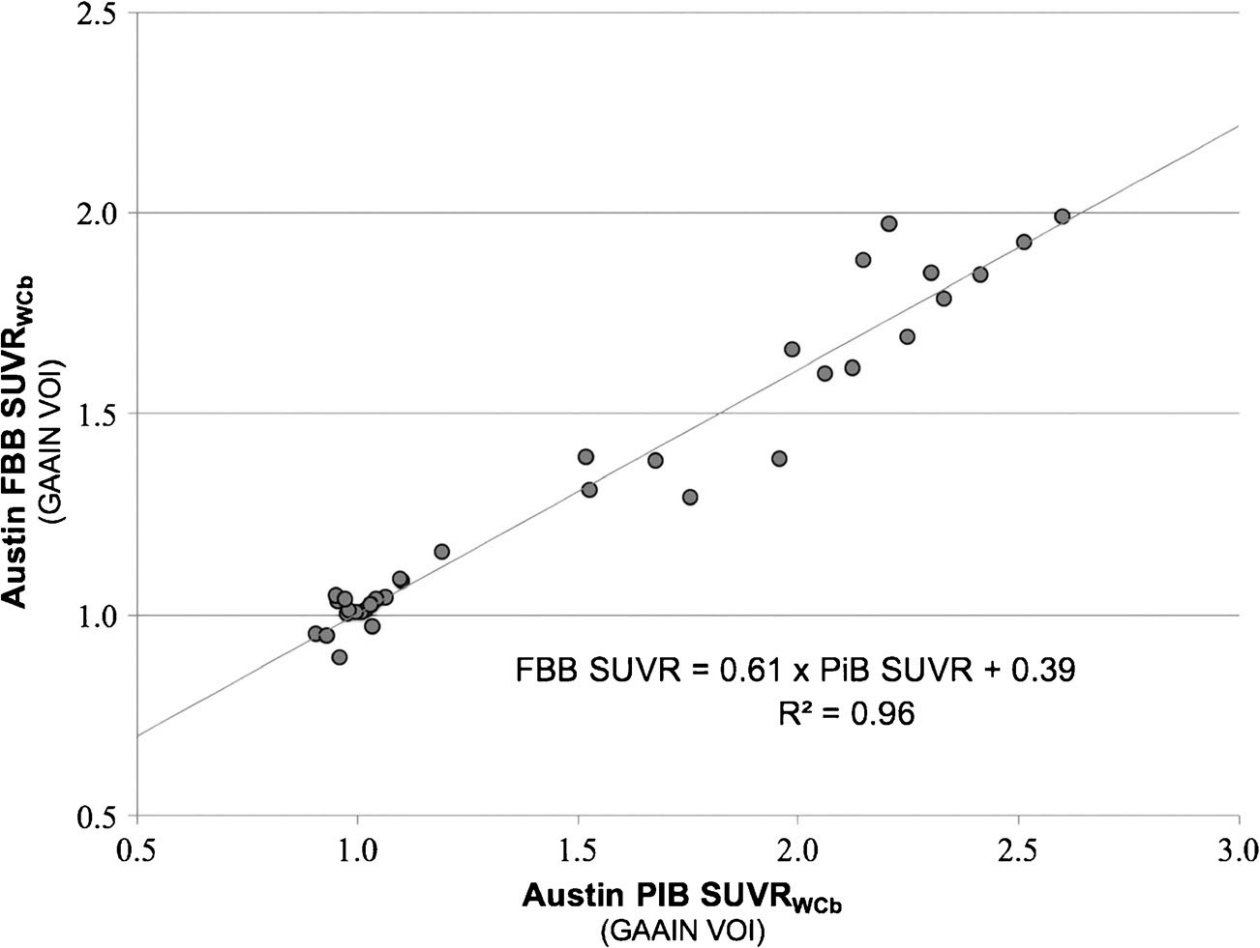
Plot of the paired ^11^C-PiB SUVR_WCb_ and ^18^F-FBB SUVR_WCb_ for each subject calculated by the standard Centiloid method with the standard large single cortical region of interest and the whole cerebellum as reference region

Validation of local implementation of the standard CL method on the PiB scans obtained from the CL GAAIN website gave a linear fit of CL_Austin_ = CL_GAAIN_ – 0.07, with *R* ^2^ = 0.9999. The fit exceeded the minimum specified acceptance criteria (i.e. *R* ^2^ > 0.98, slope 0.98–1.02, intercept between −2 and +2) [18], confirming that local implementation of the standard CL method was accurate. The locally acquired paired PiB and FBB images were then analysed with the standard CL templates and method and demonstrated excellent linear correlation: SUVR_FBB_ = 0.61 × SUVR_PiB_ + 0.39, *R* ^2^ = 0.96 (Fig. 4). The strong correlation satisfied the CL method criteria of a correlation between tracers of *R* ^2^ > 0.70 to be valid for the CL process.

SUVR_FBB_ was converted to the equivalent SUVR_PiB_ using the above equation and the results were then transformed to CL units using the published equation for conversion of PiB standard SUVR to CL units. The linear equation required to directly convert FBB binding to CL units was then CL = 153.4 × SUVR_FBB_ − 154.9. The mean and variance of the FBB and PiB CL units in the young adult normal subjects were −0.32 ± 6.81 and −1.08 ± 3.48, respectively, yielding a variance ratio of 1.96 (SD_FBB_/SD_PiB_).

## Discussion

The study demonstrated that the Aβ imaging tracer FBB has binding properties that allow conversion of SUVRs to CL units by linear transformation. The equation CL = 153.4 × SUVR_FBB_ – 154.9 enables FBB SUVRs from scans acquired from 90 to 110 min after injection of tracer and analysed by the standard CL method to be converted to CL units without the need to acquire paired PiB scans. This linear equation may now be applied to FBB scans obtained at other sites to derive CL values when FBB SUVRs have been calculated by the standard CL method. The CL method uses widely available, public domain programs to facilitate this process and makes available a standard dataset for method validation. The FBB, PiB and MRI scans used in this analysis and the results have been uploaded to the GAAIN website (http://www.gaain.org) to serve as a validation dataset for other users.

An additional advantage of the standard CL method is that it provides a mechanism to compare Aβ tracers against PiB in a standard manner. The tight correlation between FBB and PiB binding (*R* ^2^ = 0.96) indicates that for clinical and research applications FBB will perform well compared with PiB. The slope of this plot (SUVR_FBB_ = 0.61 × SUVR_PiB_ + 0.39) reveals that, as for other FDA-approved Aβ tracers [28], FBB binding to Aβ has a narrower dynamic range and this is reflected in the equations for converting between PiB and FBB uptake values: CL = 93.7 × SUVR_PiB_ – 94.6 and CL = 153.4 × SUVR_FBB_ – 154.9, respectively. The variation in FBB binding was higher than that in PiB binding in the young individuals who we expected to have no Aβ in the brain. The significance of these findings is unclear. While they might indicate less ability to detect subtle changes in Aβ burden, comparison between radioligands should consider applying other measures such as test–retest variability, and correlation with histopathology that are beyond the scope of this paper. Indeed, FBB showed excellent results in a phase III post mortem study of detection of AD [27], and has been shown to detect subtle changes in Aβ over time in a population of subjects with MCI [26], matching the changes found using PiB [19].

The CL method allows conversion of the uptake values of any Aβ tracer to a unified scale and it is expected that this information for the other tracers approved by EMA and FDA, florbetapir and flutemetamol, will be published in the near future. Ideally, FBB images obtained at a particular site will be reprocessed using the standard CL method and the conversion equation provided in this report will be applied to quantify the studies in CL units. However, it should be possible to convert a global SUVR determined by a local method to CL units by analysing the FBB dataset that we have placed on the GAAIN website where, provided there is good correlation with the standard CL method for these images (*R* ^2^ > 0.7) [18], a further linear transformation for locally preferred analysis methods can be added to permit expression in CL units.

It may be that differences between PET systems and reconstruction methods have an effect on the use of the conversion equation at other sites. Further work is needed to determine if this could be an issue for the CL method, and whether equipment-specific equations are needed. The increasing availability of standardized results raises the issue of thresholds for research and clinical application. One approach is to define the upper limit for a normal range (i.e. negative scan) as two standard deviations above the mean value determined in young normal subjects. This provides a value of 7 CL units for PiB and 14 CL units for FBB based on the variance from the ten young normal subjects in this study. However, this is well below the equivalent CL value for the visual cut-off point or PiB SUVR used in most research studies and well below the SUVR value of 1.478 obtained from ROC analysis in the FBB phase III study [27] that best matched the histopathological cut-off criteria for AD in an elderly end-of-life population. Of note, in that post mortem correlation study, the standard CL SUVR method was not used as the cortical regions differed, and the cerebellar cortex was used as the reference region. We estimate that this phase III SUVR threshold is approximately 25–30 CL. To resolve these issues, standardized CL analyses of the clinical phase III and other post mortem study scans are required to set CL unit thresholds for the histopathological classification of Aβ plaques proposed by the Consortium to Establish a Registry for Alzheimer’s Disease, i.e. none, sparse, moderate and frequent plaque levels.

### Conclusions

In summary, implementation of the CL method for quantification of Aβ PET imaging results is an important step towards better clinical and research use of Aβ imaging. It will allow the use of multiple Aβ tracers in studies such as multicentre trials of anti-Aβ therapies, and provide better diagnostic and prognostic data to clinicians by application of cut-off values that are applicable to all Aβ scans. Aβ burden measured in terms of FBB uptake can now be expressed in CL units. The dataset supporting the conclusions of this article is available on the GAAIN website (http://www.gaain.org).
